# Supplementary figures and images for: Calcium-responsive transactivator (CREST) toxicity is rescued by loss of PBP1/ATXN2 function in a novel yeast proteinopathy model and in transgenic flies
Source: PLoS Genet. 2019 Aug 7;15(8):e1008308. doi: 10.1371/journal.pgen.1008308 (PMC6699716; doi:10.1371/journal.pgen.1008308)

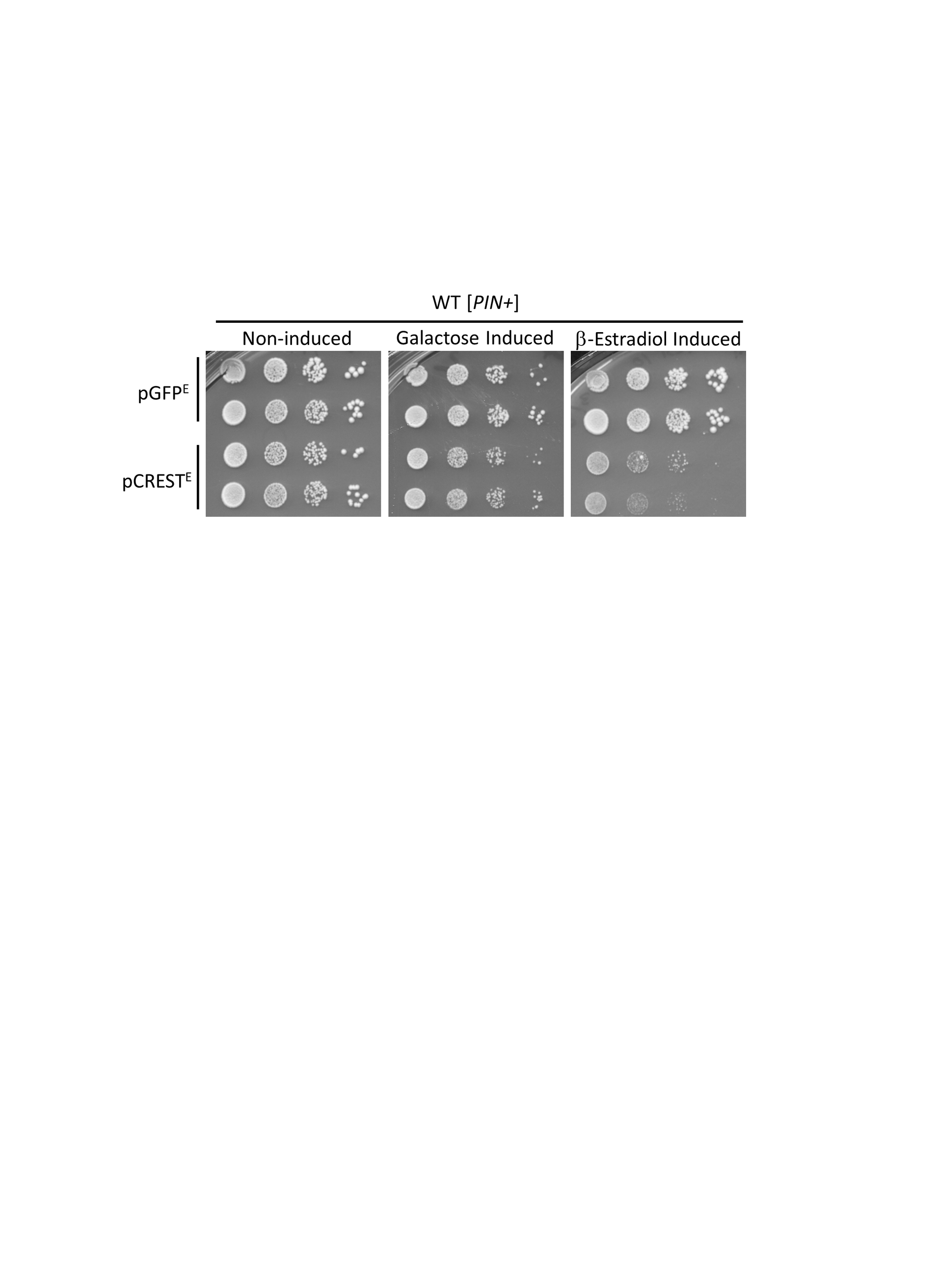

Supplement: S1 Fig — Cells transformed with pβ-est and YEpGAL-CREST-GFP (pCRESTE) or the control YEpGAL-GFP (pGFPE) were serially diluted and spotted on plasmid selective glucose (Non-induced), galactose (Galactose induced) or glucose with 2μM β-estradiol medium. (TIF) [file pgen.1008308.s001.tif]

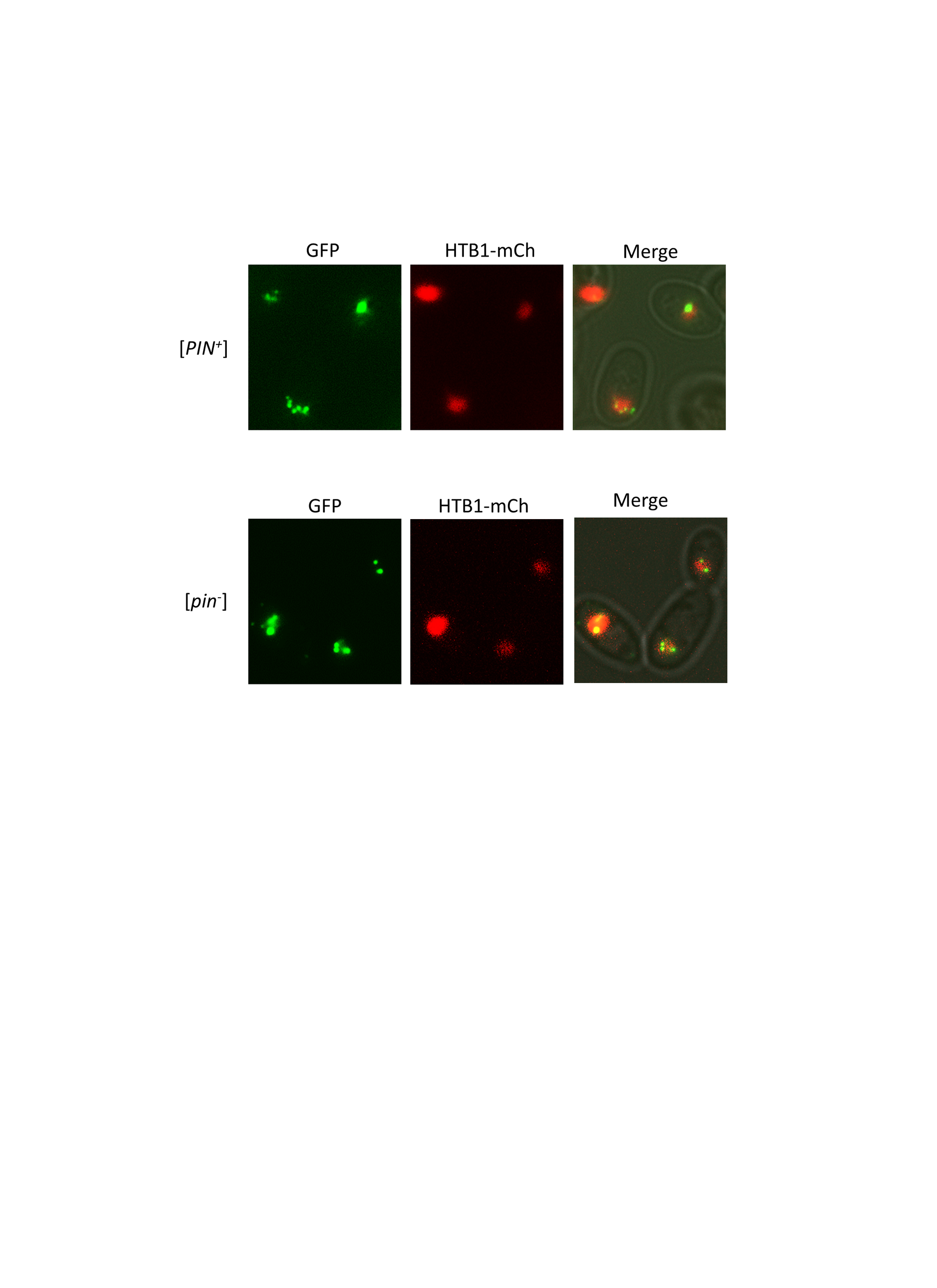

Supplement: S2 Fig — The strains L3491 and L3496 with integrated plasmid encoding ADH1-HTB1-mCh transformed with YCpGAL-CREST-GFP were grown on plasmid selective SRGal medium for 24 h and examined. (TIF) [file pgen.1008308.s002.tif]

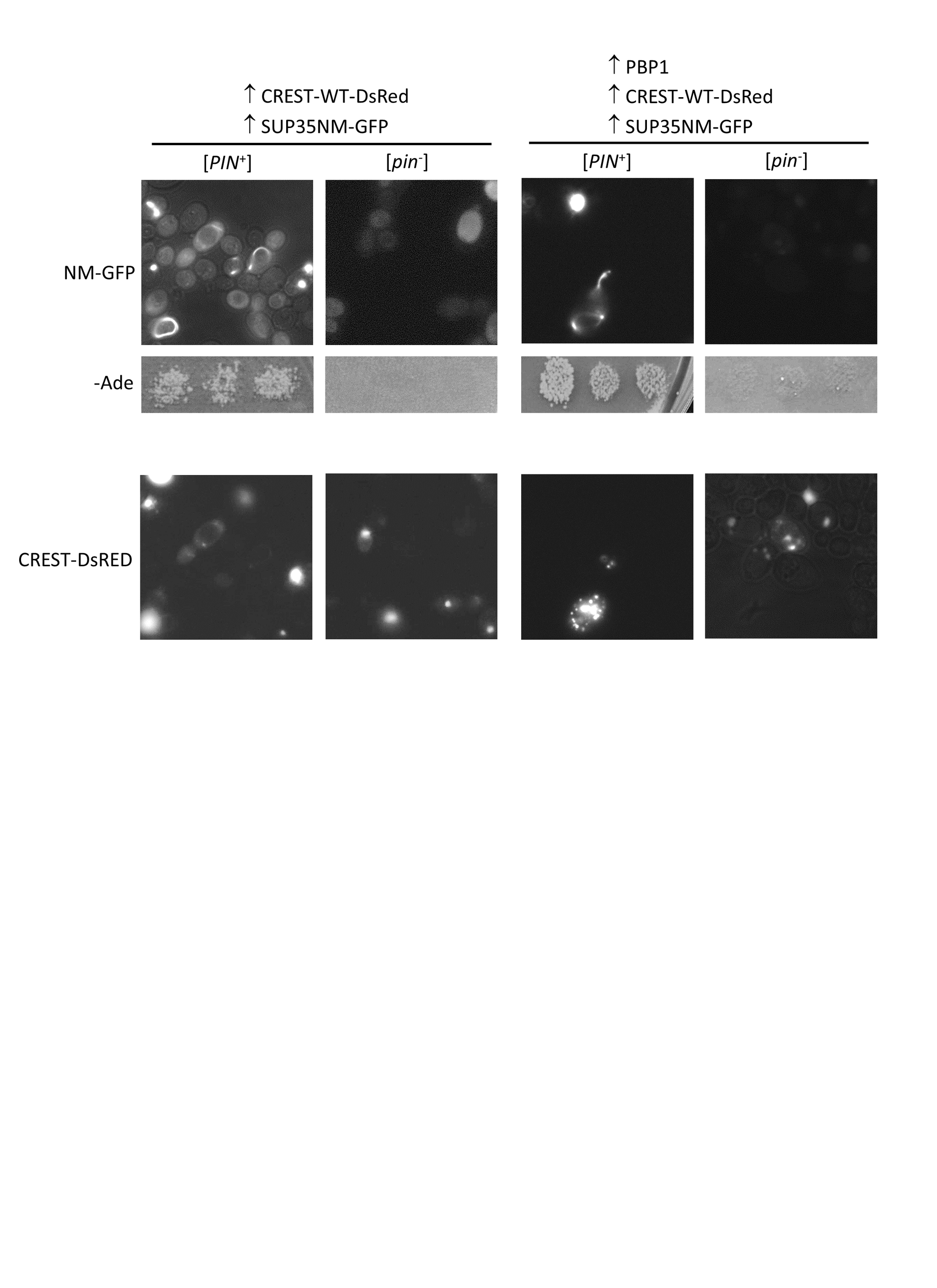

Supplement: S3 Fig — Isogenic [PIN+] and [pin-] 74D-694 strains transformed with plasmids YEpGAL-CREST-DsRED (↑CREST-DsRED) and CUP1-SUP35NM-GFP (↑SUP35NM-GFP) and YCpGAL-PBP1-EGFP (↑PBP1) were patched on plasmid selective SGal plates with 50 μM CuSO4 and grown overnight. Cells were then examined under a fluorescent microscope with a GFP filter (upper) or mCherry filter (lower) and replica-plated onto medium lacking adenine (-Ade) where only [PSI+] cells can grow. Plates were photographed after incubation. Patches of three independent transformants of [PIN+] and [pin-] are shown (middle). (TIF) [file pgen.1008308.s003.tif]

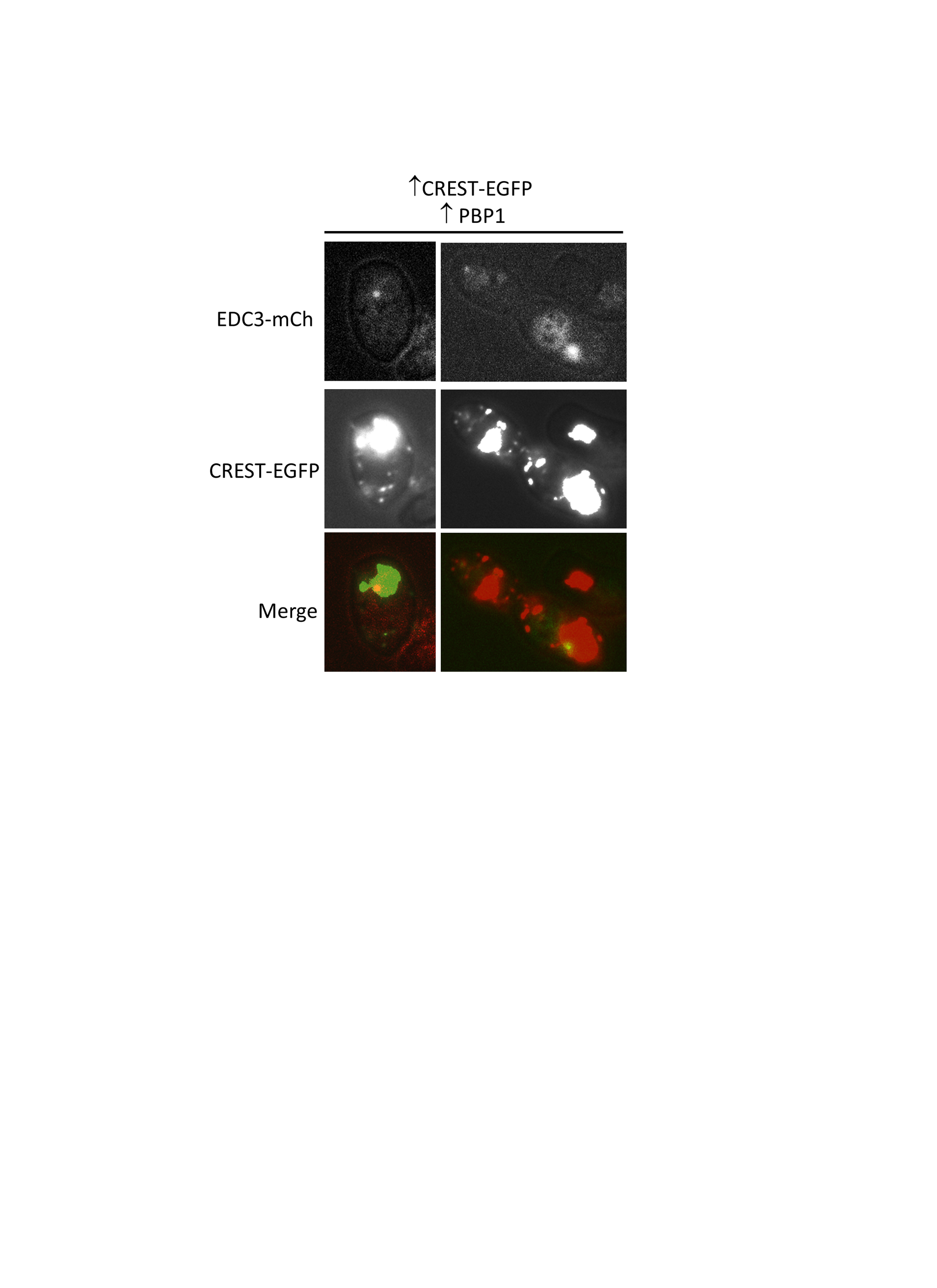

Supplement: S4 Fig — L1749 transformed with YEpGAL-CREST-GFP, YCpGAL-PBP1-TRP and either YCpDED1-mCh or YCpEDC3-mCh were grown on plasmid selective galactose media and examined. DED1 failed to form any dots while EDC3 dots were very rare in these stress-free cultures. (TIF) [file pgen.1008308.s004.tif]
